# Prediction of pathologic stage in non-small cell lung cancer using machine learning algorithm based on CT image feature analysis

**DOI:** 10.1186/s12885-019-5646-9

**Published:** 2019-05-17

**Authors:** Lingming Yu, Guangyu Tao, Lei Zhu, Gang Wang, Ziming Li, Jianding Ye, Qunhui Chen

**Affiliations:** 1 Department of Radiology, Shanghai Chest Hospital, The Affiliated Chest Hospital of Shanghai Jiaotong University, No. 241 Huaihai West Road, Xuhui District, Shanghai, 200030 China; 2 Department of Pathology, Shanghai Chest Hospital, The Affiliated Chest Hospital of Shanghai Jiaotong University, Shanghai, 200030 China; 3 Center for Statistics, Shanghai Chest Hospital, The Affiliated Chest Hospital of Shanghai Jiaotong University, Shanghai, 200030 China; 4 Center for Lung Tumor Clinical Medical, Shanghai Chest Hospital, The Affiliated Chest Hospital of Shanghai Jiaotong University, Shanghai, 200030 China

**Keywords:** Non-small cell lung cancer (NSCLC), Computed tomography (CT), Radiomics, Machine learning algorithm

## Abstract

**Purpose:**

To explore imaging biomarkers that can be used for diagnosis and prediction of pathologic stage in non-small cell lung cancer (NSCLC) using multiple machine learning algorithms based on CT image feature analysis.

**Methods:**

Patients with stage IA to IV NSCLC were included, and the whole dataset was divided into training and testing sets and an external validation set. To tackle imbalanced datasets in NSCLC, we generated a new dataset and achieved equilibrium of class distribution by using SMOTE algorithm. The datasets were randomly split up into a training/testing set. We calculated the importance value of CT image features by means of mean decrease gini impurity generated by random forest algorithm and selected optimal features according to feature importance (mean decrease gini impurity > 0.005). The performance of prediction model in training and testing sets were evaluated from the perspectives of classification accuracy, average precision (AP) score and precision-recall curve. The predictive accuracy of the model was externally validated using lung adenocarcinoma (LUAD) and lung squamous cell carcinoma (LUSC) samples from TCGA database.

**Results:**

The prediction model that incorporated nine image features exhibited a high classification accuracy, precision and recall scores in the training and testing sets. In the external validation, the predictive accuracy of the model in LUAD outperformed that in LUSC.

**Conclusions:**

The pathologic stage of patients with NSCLC can be accurately predicted based on CT image features, especially for LUAD. Our findings extend the application of machine learning algorithms in CT image feature prediction for pathologic staging and identify potential imaging biomarkers that can be used for diagnosis of pathologic stage in NSCLC patients.

**Electronic supplementary material:**

The online version of this article (10.1186/s12885-019-5646-9) contains supplementary material, which is available to authorized users.

## Background

Lung cancer is one of the most frequent types of malignancy and a leading cause of cancer-associated mortality worldwide [1]. In clinical management, lung cancer can be classified in two main categories, non-small cell lung cancer (NSCLC) and small cell lung cancer, with the former occupying approximately 85% of lung cancers [2, 3]. NSCLC represents a heterogeneous group of cancers mainly composed of lung squamous cell carcinoma (LUSC) and lung adenocarcinoma (LUAD) [4, 5]. However, the 5-year survival of NSCLC remains dismal, and 70% cases are diagnosed after the onset of advanced local or metastatic disease. The prognosis varies widely according to tumor staging at diagnosis [6]. Unfortunately, merely 15% of cases are not diagnosed until late stage [7], and thus the accurate prediction of pathologic stage for patients with lung cancer is of utmost importance.

Staging plays a crucial role in the evaluation of a patient as it defines the actual extent of the disease. Pathologic tumor stage is considered a pivotal factor relating to survival in NSCLC, and the 5-year survival rates vary from 83% in pathological stage IA to 23% in stage IIIA tumors [8]. Accurate staging is conducive to developing the effective medical treatment and to predicting patient prognosis. With the widespread application and advanced imaging technology in screening and diagnosis, the pathologic stages of more tumors have been diagnosed. However, the rate of recurrence remains unsatisfactory and ranges between 15 and 30%, even after complete surgical resection [9]. Pathologic staging of LUSC and LUAD remains a challenge for the physician using individual pretreatment variables.

Recent advances in radiography, such as high-resolution computed tomography and the widespread practice of low-dose helical computed tomography (CT) for screening of tumors, have led to an increase in the early detection of NSCLC [10]. CT has been widely used as a noninvasive diagnostic modality for diagnosis, clinical staging, survival prediction, surveillance of therapeutic response in patients with NSCLC [11–13]. Tumor phenotypic differences, such as irregular shapes and heterogeneity, can be measured using radiomic features derived from CT images.

Radiomics is an emerging technique that utilizes high-throughput quantitative image features for diagnosis and prognosis [14]. Radiomics focuses on systematic quantification of the tumor phenotype by effectively extracting and analyzing massive image data [15, 16]. An increasing number of studies have suggested that CT image features have high diagnostic and predictive values in clinical pathologic staging of diseases and clinical outcomes [17–19]. For instance, in a previous research of Kim et al., CT radiomics features, including roundness and grey-level nonuniformity, were verified as predictive biomarkers of survival in LUAD [20]. Ravanelli et al. performed a texture analysis on contrast-enhanced computed tomography images in advanced LUAD and uncovered an independent predictive indicator for treatment response [21]. Nonetheless, few image features can be used for accurate prediction of pathologic stage in patients with NSCLC.

Herein, our study applied a series of machine learning algorithm, and explored potential imaging biomarkers that can be used for diagnosis and prediction of pathologic stage in NSCLC based on a CT image feature analysis.

## Methods

### Data sets

A total of 145 patients with pathologically confirmed stage IA to IV NSCLC were included in this study. The patient cohort was comprised of three datasets, including NSCLC (*n* = 87), LUAD (*n* = 24) and LUSC (*n* = 34). NSCLC samples were averagely divided into a training set and a testing set, while LUAD and LUSC data sets were used for external validation. CT images of all patients were publicly available on the cancer imaging archive (TCIA). The clinico-pathologic characteristics of patients in NSCLC, LUAD, and LUSC cohorts were shown in Table 1. The inclusion criteria were those who were newly diagnosed or treatment-naive NSCLC and pathologically confirmed stage IA to IV lung adenocarcinoma and squamous cell carcinoma, as well as had pre-treatment CT images. The exclusion criteria are the patients who were treated with surgery or chemoradiation therapy and contained incorrect staging information. The TRIPOD checklist is appended as Additional file 1: Table S1.

**Table 1:** Patient and tumor characteristics in the training and validation sets

	NSCLC (*N* = 87)	TCGA-LUAD (*N* = 24)	TCGA-LUSC (*N* = 34)	*P* value
sex				
male	58	9	19	0.033
female	29	15	15	
Overall stage				
IA	14	5	3	0.513
IB	28	5	10	
IIA	5	2	5	
IIB	22	3	9	
IIIA	10	7	4	
IIIB	3	1	2	
IV	5	1	1	

### Lesion recognition and region-of-interest segmentation by 3D-slicer software

It is well known that manual segmentation is required before extracting radiological features. All patient images were loaded and processed in the original DICOM format. We used 3D-Slicer software (https://www.slicer.org/) to load CT image files and RTSTRUCT files for mapping sub-regions of lesions. Apply the segment editor module to change the main representation from a flat outline to a binary label map. The 3D image file and the binary mask tag file are saved by the 3D-Slicer as NRRD format files for the next feature extraction step.

### Extracting features from CT images using Pyradiomics

By segmenting the region of interest of the tumor, features can be extracted and four types of imaging features (shape, intensity, texture, and wavelet) can be identified. We used pyradiomics (http://readthedocs.org/projects/pyradiomics/) which is an open source python package to perform feature extraction tasks. Some quantitative features are as follows: first-order features, shape features, gray level co-occurrence matrix (GLCM) features. In addition to the shape features, other features can be measured on the original or derived image, while the shape descriptor is independent of the gray value and is extracted from the label mask (http://pyradiomics.readthedocs.io/en/latest/ Features.html).

### Data preprocessing

First, we should confirm whether the original class distribution of NSCLC cohort was balanced. If not, over sampling would be performed by means of SMOTE algorithm, to tackle the curse of imbalanced datasets in machine learning and to achieve equilibrium of class distribution by producing a new data set. The newly-generated data sets were then split up into a training set and a testing set.

### Predictive modeling and feature selection

Considering some redundant and irrelevant features that may influence classification accuracy of the prediction model, we calculated the importance value of CT image features by means of Random Forest algorithm, and then selected optimal features in accordance with feature importance (mean decrease gini impurity > 0.005) for modeling. Random forest is a tree-based ensemble learning method for regression and classification, developed by Leo Breiman [22]. It is widely applied in medicine, and has proven to be an easy-to-use and highly accurate predictive method [23]. From the methodological perspective of feature selection, random forest is a kind of embedded feature selector which can automatically produce the relative importance of features during the model training process. Here, the classification accuracy of the random forest was evaluated using out of bag (OOB) error, which is an unbiased estimate of random forest generalization error. We used the python module scikit-learn to perform all the above modelling process using default parameters. In view of the limited sample size of each stage, we also performed all the aforementioned analysis on binarized stage of early (stage I/II) and late (stage III/IV).

### Classification accuracy of prediction model

To evaluate the performance of prediction model in training and testing sets, receiver operating characteristics (ROC) curves were plotted to display classification performance in the testing set and the external validation set. The ROC curve is a comprehensive index that reflects false positive rate and true positive rate of continuous variables. The area under the curve (AUC) was an evaluation measure for model performance.

In addition, confusion matrix was applied to examine whether there is a consistency between the predicted and actual results. Confusion matrix is a useful tool to evaluate the performance of classifiers in their ability to classify multi-classed objects in addition to ROC curves. In this study, we focused on the generalization properties of learning algorithm for multiclass classification problems and used the confusion matrix of a classifier as a measure of its quality. We also used accuracy score, the ration of number of correctly classified samples to the number of all the samples, to evaluate the model predictive performance. Finally, we computed a new model by using original features, the accuracy score of which could be calculated according to the chosen optimal features.

### Assessment of prediction model using precision-recall curves

In addition to evaluating accuracy of prediction model using ROC curves, Precision-Recall metric was also employed to estimate the output quality of the classifier. Precision-Recall curves is more informative when evaluating binary classifier on imbalanced datasets with performance measures such as precision and recall metrics. A high area under the curve of a precision-recall curve can be detected with either high precision or high recall, which also suggests a low false positive rate or a low false negative rate. High scores for both show that the classifier is returning accurate results (high precision), as well as returning a majority of all positive results (high recall). Moreover, the higher f1-score, the more stable the classification model.

Considering the limitation of single metrics-precision, recall and f1-score, we decided to adopt average precision score and precision-recall to each class to assess the overall capacity. Here, average precision (AP) is used to measure the accuracy of the classifier using weighted mean of precisions achieved at each threshold. Furthermore, the output would be binarized if the precision-recall curve and average precision were extended to multi-class or multi-label classification. The precision-recall curve can be plotted through considering each element of the label indicator matrix, which is considered a binary prediction (micro-averaging).

## Results

### Clinico-pathologic characteristics

A total of 145 were pathologically diagnosed with NSCLC, including LUSC, LUAD, or other subtypes of NSCLC. In the training/testing sets with 87 NSCLC patients, there were 58 male patients and 29 female ones. Furthermore, the LUAD cohort comprised of 24 patients and the LUSC cohort consisting of 34 ones were separately used for the external validation of the model. As shown in Table 1, no discrepancy was detected between the training/testing sets and the validation set in gender (*P* = 0.14), while there was a significant difference found between three cohorts in terms of clinico-pathologic stages (*P* = 0.02). Clinical information for NSCLC patients from TICA database was provided in Additional file 2: Table S2.

### Identification of imaging biomarkers

**Fig. 1:**
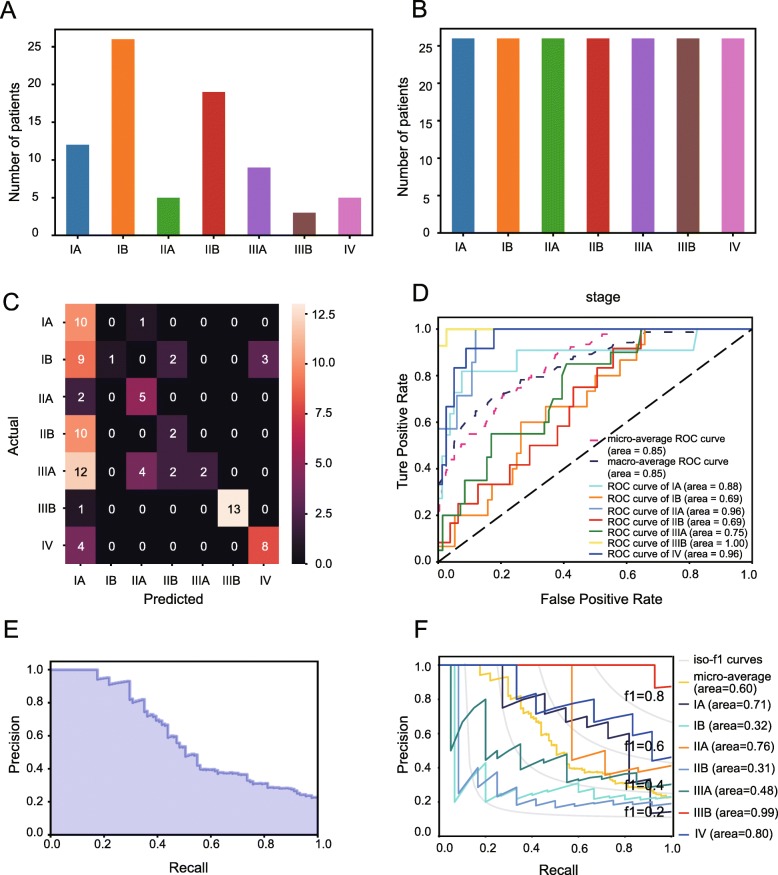
Performance assessment of prediction model in training and testing sets. **a** The imbalanced class distribution of NSCLC samples. **b** The final class distribution of NSCLC samples after equilibrium processing. **c** Confusion matrix was used to examine whether there is a consistency between the actual and the predicted results in NSCLC cohort. **d** Receiver operating characteristic (ROC) curve analysis for the prediction of the pathologic stages in NSCLC cohort. The corresponding reference groups are all the other stages patients. **e** Average precision score of prediction model in NSCLC cohort, micro-average over all classes: AP = 0.60. **f** Extension of precision-recall curve to multi-classes in NSCLC cohort

Through observation on data of each group, we confirmed the imbalanced class distribution of original NSCLC samples in the training set (Fig. 1a), and then conducted over sampling by using random oversampling. Another machine learning algorithm, SMOTE, was subsequently employed to generate a new balanced data set for the following analyses (Fig. 1b).

Given that some redundant or irrelevant features in the new data set may exert an influence on the classifying effects of the model, the importance value of CT image features was first calculated by means of Random Forest algorithm (Table 2), followed by selection of the optimal features based on each feature importance (mean decrease gini impurity > 0.005). In total, nine image features were chosen for modeling. These features are wavelet-HHH_firstorder_RootMeanSquared, log-sigma-2-0-mm-3D_firstorder_RootMeanSquared, wavelet-HHL_glcm_InverseVariance, wavelet-HHL_glcm_Idn, wavelet-HHL_firstorder_Variance, wavelet-HHL_glszm_SmallAreaHighGrayLevelEmphasis, wavelet-HHL_glcm_InverseVariance, wavelet-HHL_glcm_Imc1, wavelet-HHL_glrlm_LongRunLowGrayLevelEmphasis, respectively. All the features are numerical features. There nine features can be utilized as the stage-predictive image biomarkers. Several features are easy to interpret. Root mean squared (RMS) is the square-root of the mean of all the squared intensity values. It is a measure of the magnitude of the image values. As for inverse difference normalized (IDN), it is a measure of the local homogeneity of an image. Small Area High Gray Level Emphasis (SAHGLE) measures the proportion in the image of the joint distribution of smaller size zones with higher gray-level values. Long Run Low Gray Level Emphasis (LRLGLE) measures the joint distribution of long run lengths with lower gray-level values. Ultimately, the classification accuracy of the models was evaluated using OOB error, with 0.81 of original random forest model and 0.86 of limited feature model.

**Table 2:** Feature importance

Feature	Importance
wavelet-HHH_firstorder_RootMeanSquared	0.007136
log-sigma-2-0-mm-3D_firstorder_RootMeanSquared	0.006829
wavelet-HHL_glcm_InverseVariance	0.006782
wavelet-HHL_glcm_Idn	0.006155
wavelet-HHL_firstorder_Variance	0.005531
wavelet-HHL_glszm_SmallAreaHighGrayLevelEmphasis	0.00533
wavelet-HHL_glcm_InverseVariance	0.005291
wavelet-HHL_glcm_Imc1	0.005072
wavelet-HHL_glrlm_LongRunLowGrayLevelEmphasis	0.005063
wavelet-HHL_glcm_Idmn	0.004948
wavelet-HHL_glrlm_GrayLevelVariance	0.004713
wavelet-HHL_glszm_LargeAreaLowGrayLevelEmphasis	0.004235
wavelet-HHL_glcm_Idm	0.004189
wavelet-HHL_glrlm_ShortRunHighGrayLevelEmphasis	0.004122
wavelet-LLL_glrlm_LongRunHighGrayLevelEmphasis	0.003965
wavelet-HLH_glcm_JointEnergy	0.003955
wavelet-HHL_gldm_LargeDependenceEmphasis	0.003925
original_glszm_ZoneVariance	0.003886
log-sigma-2-0-mm-3D_glcm_ClusterProminence	0.003725
wavelet-HHL_firstorder_Median	0.003717
wavelet-HHL_gldm_SmallDependenceHighGrayLevelEmphasis	0.003683
wavelet-HHL_glrlm_LongRunHighGrayLevelEmphasis	0.003615
wavelet-HHL_glcm_DifferenceVariance	0.003579
log-sigma-4-0-mm-3D_glszm_GrayLevelNonUniformity	0.003525
wavelet-LLH_firstorder_RootMeanSquared	0.003449
wavelet-LLL_glszm_SizeZoneNonUniformityNormalized	0.003391
wavelet-HLL_glszm_GrayLevelVariance	0.003327
log-sigma-4-0-mm-3D_glrlm_ShortRunEmphasis	0.003289

### Performance evaluation of prediction model in training sets

The prediction model’s performance was first assessed in the testing sets with equilibrium of class distribution and balanced data. In terms of classification accuracy, confusion matrix results confirmed that there was a consistency between the predicted and actual results, which suggested a better performance of the model in the classification of multi-class objects (Fig. 1c). Furthermore, ROC curve analysis also verified that the model could predict and distinguish pathologic stages of NSCLC with high accuracy of 0.69 ~ 1.00 (Fig. 1d). Here, the accuracy scores of the original random forest model and limited feature model were 0.53 and 0.57, respectively.

**Fig. 2:**
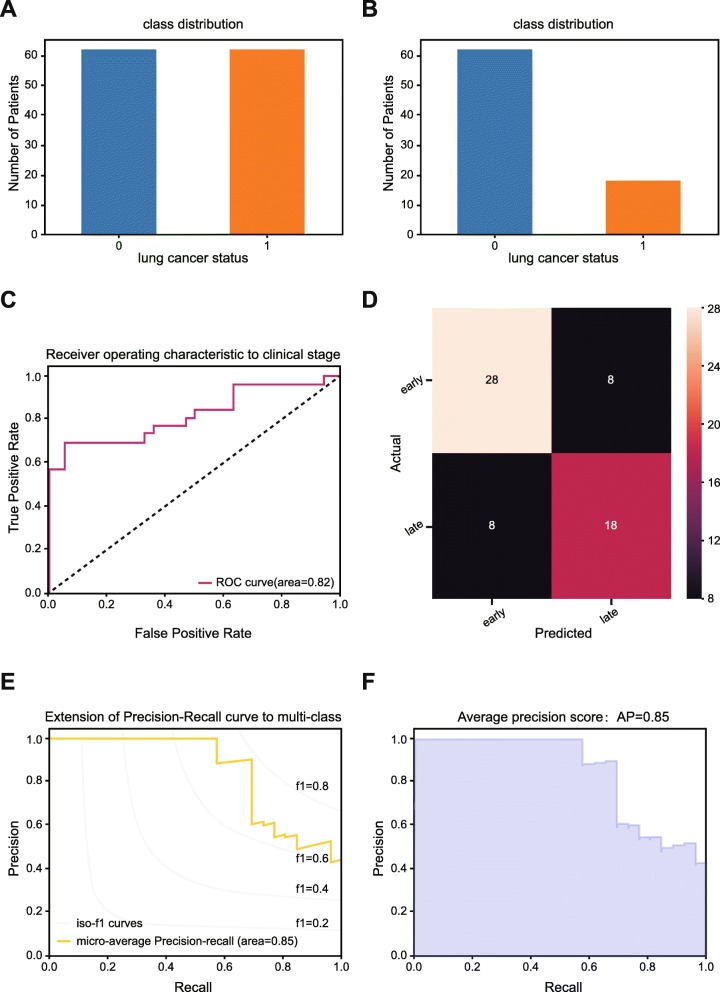
Performance assessment of prediction model in training/testing sets in binarized predictive scenario. **a** The imbalanced class distribution of NSCLC samples. **b** The final class distribution of NSCLC samples after equilibrium processing. **c** Receiver operating characteristic (ROC) curve analysis for the prediction of the pathologic stages in NSCLC cohort. **d**. Confusion matrix was used to examine whether there is a consistency between the actual and the predicted results in NSCLC cohort. **e** Precision-recall curve in NSCLC cohort. **f** Average precision score of prediction model in NSCLC cohort

In addition to accuracy score, the prediction model was also evaluated from the perspectives of average precision score and precision-recall to each class. As exhibited in precision-recall curves (Fig. 1e, f), our prediction model not only yielded a higher average precision score (AP) of 0.60 (Fig. 1e), but achieved a better diagnostic performance for pathologic stages of NSCLC in terms of the extension of precision-recall curve to multi-classes (Fig. 1f). Corresponding results of binarized stage scenario is depicted in Fig. 2.

### Performance evaluation of prediction model in external validation sets

**Fig. 3:**
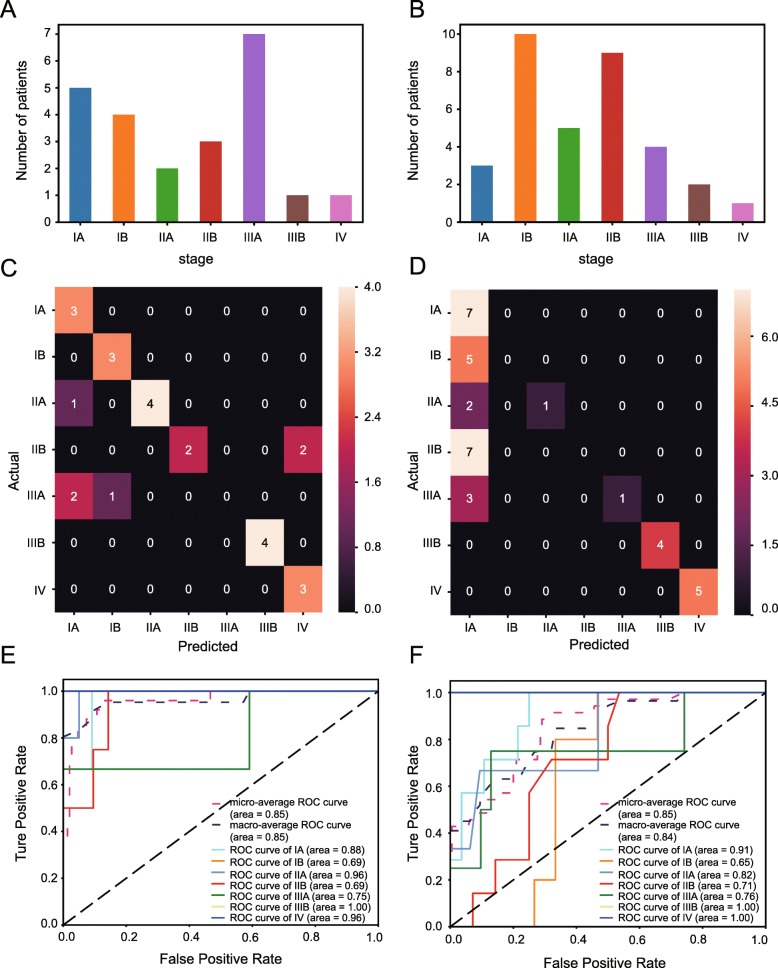
Performance assessment of prediction model in validation sets. **a** The class distribution of samples in LUAD dataset. **b** The class distribution of samples in LUSC dataset. **c** and **d**. Confusion matrix was used to determine whether there is a consistency between the actual and the predicted results in LUAD (**c**) and LUSC (**d**). **e** and **f**) Receiver operating characteristic (ROC) curve analysis for the prediction of the pathologic stages in LUAD (**e**) and LUSC (**f**). The corresponding reference groups are all the other stages patients

**Fig. 4:**
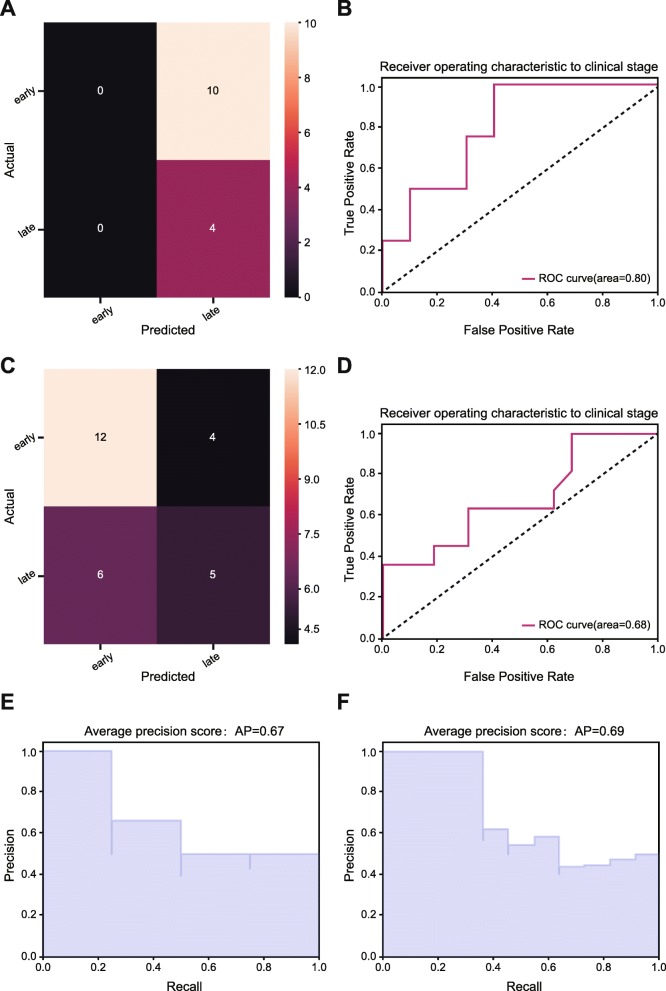
Performance assessment of prediction model in validation sets. in binarized predictive scenario. **a** & **c** Confusion matrix was used to determine whether there is a consistency between the actual and the predicted results in LUAD (up) and LUSC (down). **b** & **d** Receiver operating characteristic (ROC) curve analysis for the prediction of the pathologic stages in LUAD (up) and LUSC (down). **e** Average precision score of prediction model in LUAD. **f** Average precision score of prediction model in LUSC

In addition to the internal testing above, we also performed an external validation for the performance of the prediction model by using LUAD and LUSC data sets without preprocessing or equilibrium of class distribution. The original class distribution of samples in the LUAD cohort (Fig. 3a) and LUSC cohort (Fig. 3b) was displayed in Fig. 4. The machine learning algorithms in the external validation were same as those used in testing sets. In terms of the classifier performance in the LUAD data set, confusion matrix and ROC curves both indicated a high classification accuracy of the model, with AUC value of 0.69 ~ 1.00 (Fig. 3c, d). Likewise, we also re-confirmed the consistency between the predicted and actual results in the LUSC data set (Fig. 3e). ROC curves also revealed that the model could distinguish the pathologic stages of LUSC with high accuracy of at least 65% (Fig. 3f).

**Fig. 5:**
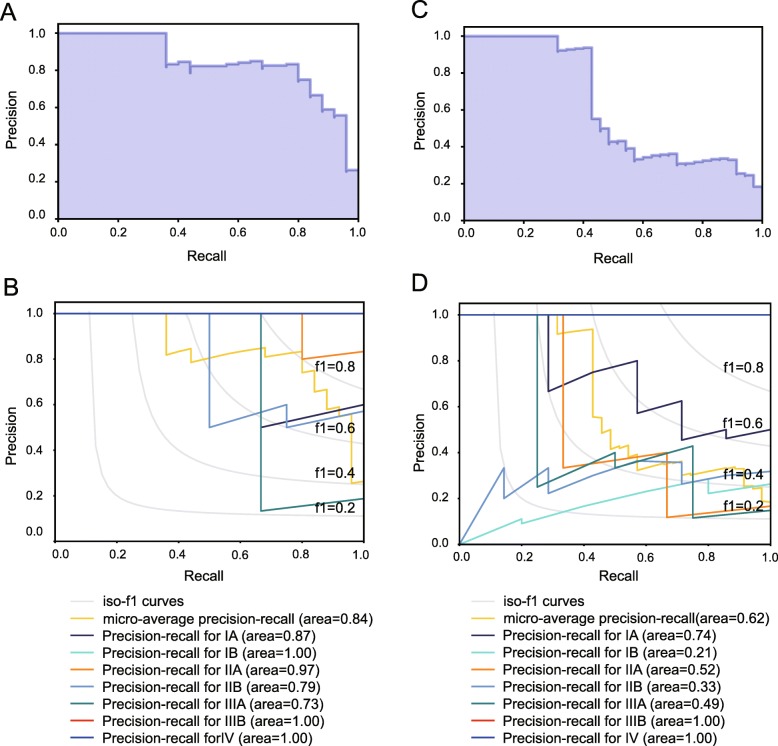
Performance assessment of prediction model in validation sets in terms of precision and recall score. **a** Average precision score of prediction model in LUAD, micro-average over all classes: AP = 0.84. **b** Extension of precision-recall curve to multi-classes in LUAD. The corresponding reference groups are all the other stages patients. **c** Average precision score of prediction model in LUSC, micro-average over all classes: AP = 0.62. **d** Extension of precision-recall curve to multi-classes in LUSC. The corresponding reference groups are all the other stages patients

For average precision score and precision-recall to each class of the model, our precision-recall curves in the external validation set presented that the prediction model not only achieved higher average precision scores in both LUAD (AP = 0.84) and LUSC (AP = 0.62) (Fig. 5a, c) but yielded a better differentiation performance for pathologic stages of LUAD and LUSC in terms of the extension of precision-recall curve to multi-classes (Fig. 5b, d). Taken together, our prediction model that incorporated nine image features could predict and differentiate the pathologic stages of NSCLC accurately, and the predictive accuracy of the model in LUAD outperformed that in LUSC. Corresponding results of binarized stage scenario is depicted in Fig. 4. In addition, the association between Radiomic Score and TNM factors was separately evaluated as shown in Additional file 3: Table S3, and similar results were found in the NSCLC dataset.

## Discussion

The present study revealed that image features extracted from CT scans was correlated with pathologic stage of patients with NSCLC. Our study further explored the function and application of machine learning in CT image feature analysis for pathologic staging, meanwhile, unveiled potential imaging biomarkers that can be used for diagnosis and prediction of pathologic stage in NSCLC. Ultimately, our prediction model that incorporated nine optimal characteristics was validated to be significantly effective in the prediction of lung cancer subtypes and pathologic tumor stages of LUAD and LUSC.

In recent years, radiomics plays an emerging role in cancer research, imaging biomarkers and clinical management. An increasing number of image characteristics have been reported to have high predictive and diagnostic values in NSCLC [12, 24]. For instance, Coroller et al. corroborated that CT-based radiomic signature could predict distant metastasis in lung adenocarcinoma [25]. Ko et al. unraveled the predictive value of 18F-FDG PET and CT morphologic features for recurrence in pathological stage IA non-small cell lung cancer [26]. Chen et al. demonstrated that a radiomics signature is a potentially useful imaging biomarker for differentiating low from high DTD in patients with NSCLC based on contrast-enhanced computed tomography imaging [27]. With continuous advances in imaging technology and researches, some radiomics features are also applied to the prediction of pathologic stage in lung cancer. Tsutani et al. identified predictors of pathologic lymph node involvement in clinical stage IA lung adenocarcinoma [28]. In a clinico-pathologic study performed by Kaira et al., fluorine-18-alpha-methyltyrosine positron emission tomography was validated to be instrumental in diagnosis and staging of lung cancer [29]. Herein, we first conducted an equalization processing for imbalanced data sets, and then determined nine optimal image characteristics that may be related to the pathologic stages of LUAD and LUSC by means of Random Forest. Our prediction mode was validated internally and externally by means of multiple machine learning algorithms.

Radiomics applies machine learning algorithms to quantitative imaging data to characterize the tumour phenotype and predict clinical outcome. Recent breakthroughs in deep learning with applications in radiology, such as lung nodule malignancy classification, pathologic stage prediction and lymph node detection, have been instrumental in identifying disease-specific imaging biomarkers and improving the diagnostic performance [30–32]. For instance, Zhang et al. also unveiled optimal machine-learning algorithms for the radiomics-based prediction of local failure and distant failure in advanced nasopharyngeal carcinoma, which could enhance the applications of radiomics in precision oncology and clinical practice [33]. Furthermore, Ferreira et al developed and validated a radiomics signature that can be used for histopathological subtype diagnosis and metastatic prediction of lung cancer based on machine learning [34]. In this study, ROC curves and confusion matrix were employed for assessment on the classification accuracy of the prediction model, meanwhile, we also estimated the output quality of the classifier was also measured comprehensively using Precision-Recall metric. The prediction model that incorporated nine image features exhibited a high classification accuracy (all AUC > 0.70), precision and recall scores (AP = 0.60) in the training sets, while the predictive accuracy of the model in LUAD (AP = 0.84) was much higher than that in LUSC (AP = 0.62) in the external validation. The results above also imply that there exists a large difference between LUAD and LUSC in terms of CT image features, and hence the two subtypes of NSCLC may be differentiated and predicted based on the difference of image features.

Nevertheless, it is noteworthy that there are some limitations in our radiomics analysis. Although our prediction model could be used for the precise tumor staging of lung cancer, some deviations may exist due to limited sample size. Moreover, the imbalanced data sets were subjected to equalization processing, while there are still some deficiencies, and thus a larger cohort would be needed for the further validation of the model. Furthermore, despite our focus is the staging of NSCLC, we still lack the CT images of healthy volunteers to be negative controls.

## Conclusions

In conclusion, it is the first time that the significance of radiomics features in prediction of pathologic stages of NSCLC has been studied. Nine optimal image features were identified as predictive and diagnostic biomarkers for pathologic stages of NSCLC. Using multiple machine learning algorithms, our prediction model has been verified to effectively predict the tumor stages of NSCLC, especially for LUAD. Our findings not only extend the application of machine learning algorithms in CT image feature prediction for pathologic staging, but identify potential imaging biomarkers that can be used for diagnosis and prediction of pathologic stage in NSCLC.

## Additional files

Additional file 1: TRIPOD Checklist: Prediction Model Development. (DOCX 88 kb)

Additional file 2: **Table S2.** Clinical information of NSCLC patients from TCIA database. (XLSX 15 kb)

Additional file 3: **Table S3.** Association between Radiomic Score and TNM factors. (XLSX 11 kb)

## Abbreviations

AP: Average precision
AUC: Area under the curve
CT: Computed tomography
GLCM: Gray level co-occurrence matrix
IDN: Inverse difference normalized
LRLGLE: Long Run Low Gray Level Emphasis
LUAD: Lung adenocarcinoma
LUSC: Lung squamous cell carcinoma
NSCLC: Non-small cell lung cancer
OOB: Out of bag
RMS: Root mean squared
SAHGLE: Small Area High Gray Level Emphasis
TCIA: The cancer imaging archive
